# Population transcriptomics: a novel tool for studying genetic diversity in human populations under normal and pathological conditions

**DOI:** 10.18699/vjgb-25-76

**Published:** 2025-09

**Authors:** A.A. Babovskaya, E.A. Trifonova, V.A. Stepanov

**Affiliations:** Research Institute of Medical Genetics, Tomsk National Research Medical Center of the Russian Academy of Sciences, Tomsk, Russia; Research Institute of Medical Genetics, Tomsk National Research Medical Center of the Russian Academy of Sciences, Tomsk, Russia; Research Institute of Medical Genetics, Tomsk National Research Medical Center of the Russian Academy of Sciences, Tomsk, Russia

**Keywords:** populations, RNA-seq, next-generation sequencing (NGS), gene expression, transcriptome, популяции, RNA-seq, массовое параллельное секвенирование, экспрессия генов, транскриптом

## Abstract

Genetic mechanisms regulating gene expression encompass complex processes such as transcription, translation, epigenetic modifications, and interactions of regulatory elements. These mechanisms play a crucial role in shaping phenotypic diversity in humans. High-throughput technologies, such as expression microarrays and next-generation sequencing (NGS), have enabled precise analysis of transcripts for thousands of genes genome-wide. These methods have enabled researchers to measure gene expression levels in various tissues and cells and to gain deeper insights into previously inaccessible biological processes. Numerous studies show that gene expression varies significantly among individuals. However, there are also notable differences between populations from different continental groups, driven by genetic, epigenetic, environmental factors, and natural selection. Furthermore, disease states represent an important factor influencing gene activity, as they can significantly alter the transcriptomic profiles of individual cells. In this context, comparative population genetic studies help uncover the molecular mechanisms underlying complex phenotypic traits and identify population-specific features of transcriptomic profiles in both health and disease. However, despite significant progress in this field, many aspects remain underexplored. Specifically, the distribution of gene expression variability among populations, the degree of research coverage for specific ethnic groups, the spectrum of biological materials used, and the contribution of population affiliation to observed differences in gene expression during pathological conditions require further investigation. This review presents an overview of contemporary research focused on analyzing variability in expression profiles across different human populations. It summarizes findings from individual studies, outlines the advantages and limitations of the methods employed, highlights key research directions in population transcriptomics, and discusses potential practical applications of the data obtained.

## Abstract (ru)

Генетические механизмы, регулирующие экспрессию генов, включают в себя сложные процессы: транскрипцию, трансляцию, эпигенетические модификации и взаимодействие регуляторных элементов. Они играют ключевую роль в формировании фенотипического разнообразия человека. Благодаря внедрению высокопроизводительных технологий, таких как экспрессионные микрочипы и массовое параллельное секвенирование (NGS), стало возможным с высокой точностью анализировать транскрипты на уровне тысяч генов по всему геному. Эти методы позволили ученым не только определять уровни экспрессии генов в различных тканях и клетках, но и глубже изучать биологические процессы и феномены, которые ранее оставались недоступными для анализа. Так, многочисленные работы подтвердили, что, несмотря на преобладание индивидуальных различий в уровне экспрессии генов, существуют также значимые вариации между популяциями, принадлежащими к разным континентальным группам, обусловленные генетическими, эпигенетическими и средовыми факторами и действием естественного отбора. Кроме того, важным фактором, влияющим на активность генов, являются заболевания, которые могут существенно изменять транскриптом отдельных клеток. В этом контексте сравнительные популяционно-генетические исследования позволяют раскрыть молекулярные механизмы, лежащие в основе сложных фенотипических признаков, и идентифицировать популяционно-специфические особенности транскриптомных профилей как в норме, так и при патологических состояниях. Несмотря на значительный прогресс в этой области, многие аспекты остаются недостаточно изученными. В частности, распределение вариабельности экспрессии генов между популяциями, степень исследованности отдельных этнических групп, спектр используемого биологического материала, а также вклад популяционной принадлежности в наблюдаемые различия экспрессии генов при патологических состояниях требуют дальнейшего изучения. В настоящей статье представлен обзор современных исследований, посвященных анализу вариабельности экспрессионных профилей в различных популяциях человека. Обобщены результаты отдельных экспериментов, описаны преимущества и ограничения используемых методов, выделены основные направления работ в области популяционной транскриптомики, а также обозначены перспективы практического применения полученных данных.

## Introduction

The human phenotype, in both health and disease, is a complex system shaped by genomic, transcriptomic, epigenetic, metabolomic, and environmental factors. From a proteincoding DNA sequence to its functional protein product in the cell, a range of molecular mechanisms is involved, among which processes occurring at the transcriptome level play a pivotal role. The transcriptome, defined as the complete set of transcripts in a cell at a specific moment in time, is central to the mechanisms of genetic regulation of cellular function. Understanding it is essential for identifying the molecular pathways underlying various functional states, including pathological ones. Numerous studies have demonstrated variability in gene expression at the population level (Spielman et al., 2007; Storey et al., 2007; Zhang et al., 2008). This phenomenon is driven by multiple factors, including environmental conditions, dietary habits, epigenetic regulation, and more. Additionally, natural selection likely contributes to the observed differences in gene expression by shaping unique genetic profiles of populations through adaptation processes. Another factor significantly influencing gene expression patterns is the presence of pathological processes.

Extensive research has revealed substantial differences in gene expression in diseases such as diabetes, cancer, cardiovascular disorders, reproductive issues, neurological conditions, and infectious diseases (Wei et al., 2011; Allard et al., 2012; Nédélec et al., 2016; Mitchell et al., 2017). Notably, the prevalence of some of these diseases varies across populations (Kelly et al., 2017). Variability in the transcriptional activity of genes linked to disease pathogenesis, shaped by long-term adaptation and fixed in each population’s gene pool, may contribute to these interpopulation differences

To date, a significant body of data has been accumulated from various experiments, indicating the presence of interpopulation variability in human gene expression. However, the distribution of this variability across populations, the extent of research coverage for specific population groups, the range of biological materials used, and the contribution of population affiliation to the observed differences in gene expression under pathological conditions remain insufficiently explored. This review synthesizes findings from studies investigating patterns of gene expression changes across human populations, outlines the advantages and limitations of the methods employed, and highlights promising directions for future research in this field.

## Microarray technologies in studying transcriptomics variability between populations

Nowadays, various technologies have been developed for characterizing and quantifying genome-wide gene expression, including hybridization-based methods (microarrays) and sequencing approaches. Hybridization methods use fluorescently labeled cDNA to bind with high-density commercial microarrays. The widespread use of microarrays for genomewide gene expression analysis has enabled the identification of multiple levels of gene expression variability within a species: interpopulation, interindividual, and intraindividual (including intertissue and intercellular levels).

One of the earliest studies dedicated to exploring population- level variability in the human transcriptome was conducted using peripheral blood from 52 individuals across three Moroccan Amazigh (Berbers) groups with distinct lifestyles: desert nomads, rural villagers, and urban dwellers (Idaghdour et al., 2010). According to the expression profile analysis obtained via microarrays, the percentage of differentially expressed genes between the studied groups ranged from 16.4 % (desert nomads vs. rural villagers) to 29.9 % (rural villagers vs. urban dwellers). The authors attributed this primarily to the predominant influence of environmental factors on shaping transcriptome variability.

However, the majority of data confirming interpopulation differences in gene expression have been derived from lymphoblasts (Dixon et al., 2007; Göring et al., 2007) and lymphoblastoid cell lines (LCLs) collected as part of the International HapMap Project (Stranger et al., 2007). These cell lines constitute a biobank of B-lymphocytes gathered from various populations – CEU (Caucasians), CHB (Chinese), JPT (Japanese), YRI (Nigerians), and AA (African Americans) – and modified with the Epstein–Barr virus to ensure viability (Baust et al., 2017). Initial studies investigating interpopulation variability using microarrays were conducted by research groups led by B. Stranger (Stranger et al., 2007), J. Storey (Storey et al., 2007), and R. Spielman (Spielman et al., 2007). These studies focused on identifying genes with differential expression between populations of European (CEU), East Asian (CHB, JPT), and African (YRI) ancestry.

In the study by R. Spielman and colleagues, a microarray covering more than 4,000 genes was used to compare expression between Caucasians (60 CEU) and Mongoloids (41 CHB and 41 JPT). It was found that over 1,000 genes (approximately 25 %) exhibited differential expression between CEU and the combined CHB/JPT group, while only 27 genes showed differences between Chinese (CHB) and Japanese (JPT) samples (Spielman et al., 2007). Cluster analysis confirmed that samples from Chinese individuals in Los Angeles (CHLA) were more similar in expression profiles to CHB/ JPT than to CEU, indicating a characteristic expression pattern associated with Asian ancestry (Spielman et al., 2007). A later study by P. Daca-Roszak and E. Zietkiewicz aimed to identify population-specific genes between Caucasians and Mongoloids. Analysis of B-lymphocyte cell lines revealed 20 genes with interpopulation expression differences. Of the 13 genes selected with the highest fold-change (FC > 2), three (UTS2, UGT2B17, and SLC7A7) confirmed their status as differentially expressed upon validation: UTS2 showed hyperexpression in Chinese samples, while UGT2B17 and SLC7A7 were hyperexpressed in Caucasians (Daca-Roszak, Zietkiewicz, 2019).

Another microarray-based transcriptome study of lymphoblasts found that approximately 17 % of genes exhibited expression differences between Caucasians (CEU) and Negroids (YRI). Many of these genes were associated with immune responses, including cytokines and chemokines (CCL22, CCL5, CCR2, CXCR3). Functional analysis revealed an enrichment of inflammatory response categories among genes differentially expressed between CEU and YRI, supporting their role in immune and infectious diseases (Storey et al., 2007).

Population differences in gene expression between Negroids and Caucasians were further analyzed using the Affymetrix GeneChip Human Exon 1.0 ST microarray, which includes over 9,100 transcripts, on an expanded sample of 176 LCLs (87 CEU and 89 YRI). It was determined that 4.2 % of transcripts showed significant expression differences between the groups. Hyperexpression of 156 genes was observed in Caucasian (CEU) samples, while 254 genes were hyperexpressed in African (YRI) samples (Zhang et al., 2008). Subsequent functional analysis identified the involvement of these genes in processes such as ribosome assembly, antimicrobial humoral response, intercellular adhesion, mRNA catabolism, and tRNA processing. Notably, nine genes (DPYSL2, CTTN, PLCG1, SS18, SH2B3, CPNE9, CMAH, CXCR3 and MRPS7) had previously been described as differentially expressed between CEU and YRI (Storey et al., 2007).

One of the largest population studies, conducted by H. Fan’s group in 2009, encompassed 210 LCLs from four ethnic groups (CEU, CHB, JPT, and YRI) using a high-density Illumina microarray covering over 11,000 transcripts. This study aimed to investigate interindividual and interpopulation differences in gene expression on a genome-wide scale and to determine the proportion of genes contributing to each type of variability. It was found that interindividual variability was a critical component of genetic differences within populations, accounting for nearly half (43 %) of the total variability in gene expression (Fan et al., 2009). These findings align with later data from full-transcriptome studies (RNA-seq) (Hughes et al., 2015), which will be discussed below.

Notably, these studies used lymphoblastoid cell lines from the same populations, but the reported gene expression variability ranged from 8 to 38 % (see the Table). This discrepancy may be attributed to several factors, including technical variability related to cell culture conditions and biological variability caused by epigenetic modifications and adaptation to in vitro conditions (Lappalainen et al., 2013). While cell culturing offers advantages, such as minimal biomaterial requirements, high reproducibility, and well-characterized properties, it introduces a significant component of variability into the transcriptomic profile. For instance, freeze–thaw cycles, culture medium composition, and cell density can substantially affect gene expression and transcriptome architecture (Baust et al., 2017). These limitations underscore the need for cautious interpretation of data derived from cell lines.

**Table 1.**
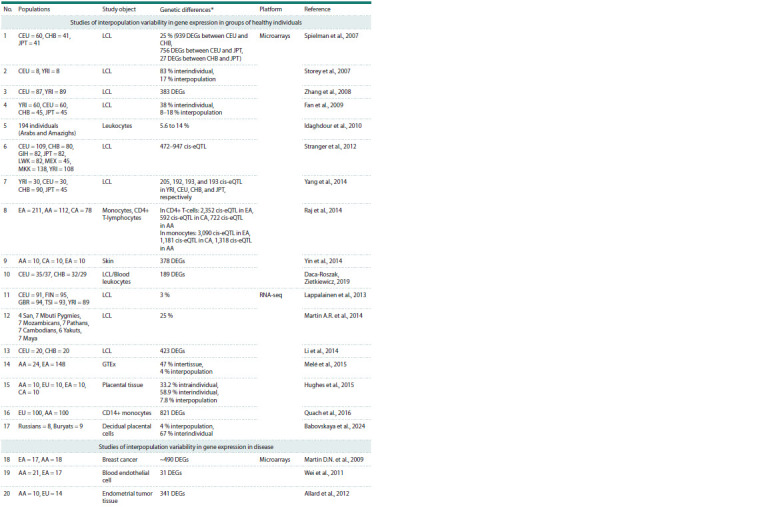
Transcriptomic studies involving two or more populations compared with each other Notе. The table uses standard population designations from the “1000 Genomes Project” (1000 Genomes Project Consortium et al., 2012) and “HapMap Project” (International HapMap Consortium, 2003): CEU – Utah residents of Central European ancestry, CHB – Chinese, GIH – Indians, JPT – Japanese, LWK – Luhya, MEX – Mexicans, MKK – Maasai, YRI – Yoruba, FIN – Finns, GBR – British, TSI – Tuscans. Introduced abbreviations: AA – African Americans, EA – Americans of European ancestry, CA – Americans of Asian ancestry, EU – Caucasians, DEGs – differentially expressed genes. * The contribution of interpopulation differences to the total variability in gene expression is provided by default

**Table 1end.**
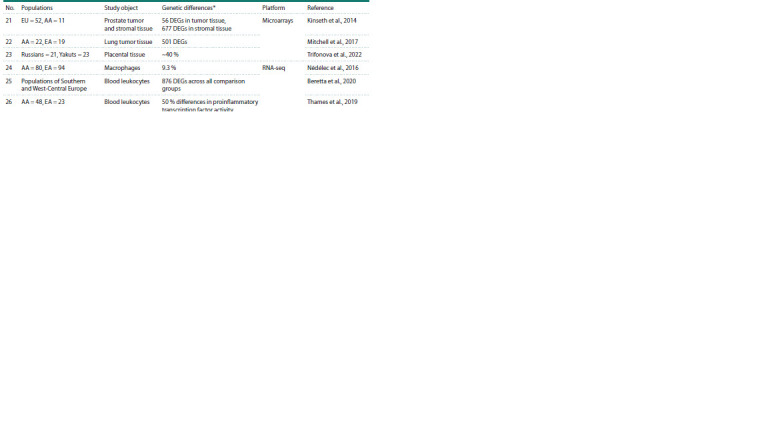
Table 1end.

Another critical factor influencing data variability is the gene expression profiling method. Despite its widespread use, microarray analysis is susceptible to the so-called “batch effect”. This effect arises from technical differences between experimental batches, such as the use of different microarray lots, analysis platforms, or variations in experimental conditions (e. g., temperature, humidity, or experiment date) (Fellenberg et al., 2006). To minimize batch effects, bioinformatics correction methods, such as Empirical Bayes methods, are employed to normalize data and reduce the impact of technical artifacts. However, such adjustments may lead to the loss of biologically significant expression differences, limiting the interpretation of results (Johnson et al., 2007).

## High-throughput sequencing technologies (NGS) in studying gene expression variability between populations

The advancement of high-throughput sequencing (NGS) technologies has provided the most comprehensive coverage of the genome and transcriptome, enabling researchers to identify key molecular pathways involved in pathological processes with high precision, detect disease biomarkers, and assess dynamic changes in gene expression in response to various stimuli. Unlike microarray profiling, NGS allows for the analysis of not only gene expression levels but also alternative splice variants, rare transcripts, and post-transcriptional modifications, significantly expanding opportunities for understanding gene regulation (Kukurba, Montgomery, 2015). Given the modern research trend toward increasing sample sizes and output data volumes, as well as several limitations imposed by microarray profiling technology (e. g., batch effects and limited genome coverage), researchers are increasingly turning to NGS methods. These approaches offer greater accuracy, reproducibility, and depth of analysis, making them preferable for studying complex biological processes (Hrdlickova et al., 2017).

In the context of studying gene expression variability across human populations, two key directions can be distinguished. The first focuses on investigating the influence of natural selection and environmental factors on shaping expression profiles. For instance, studies demonstrate that population-specific differences in gene expression are often linked to adaptations to diverse ecological conditions, such as high-altitude hypoxia, ultraviolet radiation levels, or dietary patterns (Hodgson et al., 2014). These studies help elucidate how evolutionary processes shape the transcriptomic landscape across different populations. The second direction examines differences in gene expression associated with diseases, the prevalence or clinical presentation of which varies among populations. For example, research on oncological, autoimmune and infectious diseases shows that population differences in gene expression can influence disease susceptibility, progression and response to therapy (Lappalainen et al., 2013; Quach et al., 2016; Way et al., 2016).

**Studies of interpopulation variability in gene expression in groups of healthy individuals**

Several studies in the literature have explored expression profiles across different populations using cohorts of conditionally healthy individuals. In one such study, transcriptomic variability was characterized in over 460 lymphoblastoid cell lines (LCLs) derived from individuals of an African population (YRI) and four European subpopulations (CEU, FIN, GBR and TSI) (Lappalainen et al., 2013). Interpopulation differences accounted for only a minor fraction (3 %) of the total gene expression variability. Nevertheless, the number of genes exhibiting statistically significant expression differences between the African and European populations was substantial, ranging from 1,300 to 4,300 genes depending on the European population compared. In contrast, the number of differentially expressed genes identified when comparing European subpopulations among themselves was significantly lower

In another study, gene expression was examined in 20 LCLs obtained from individuals of European (CEU) and East Asian (CHB) populations (Li et al., 2014). The analysis identified over 400 differentially expressed genes, including 132 genes with elevated expression and 291 genes with reduced expression in the CHB population compared to the CEU one

A.R. Martin and colleagues investigated transcriptomic profiles in 45 LCLs derived from seven non-European populations (Namibian San, Mbuti Pygmies from the Democratic Republic of Congo, Algerian Mozabites, Pathans from Pakistan, Cambodians from East Asia, Siberian Yakuts, and Mexican Maya). They identified 44 genes with significant differential expression across the studied populations, most of which were associated with immune pathways. The greatest interpopulation variability in expression was observed for the genes THNSL2, DRP2, VAV3, IQUB, BC038731, RAVER2, SYT2, LOC100129055, AK126080 and TTN (Martin A.R. et al., 2014).

**Fig. 1.**
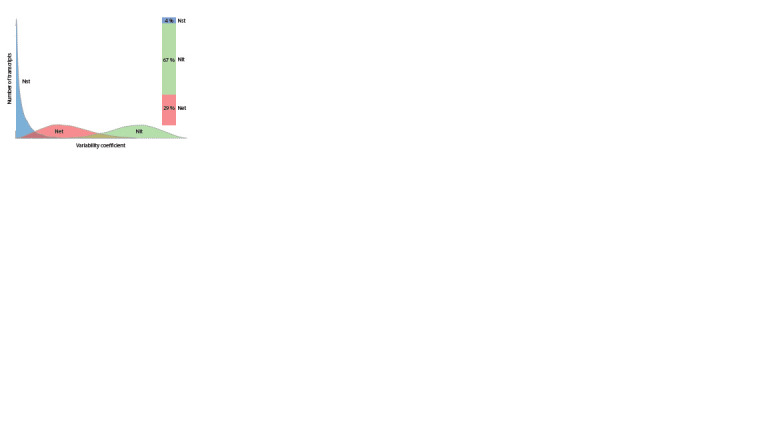
Interpopulation (Nst), interindividual (Nit), and inter-replicate (Net) differences obtained from the analysis of sums of squares for groups with physiological pregnancy.

An original approach to minimize the influence of external factors on gene expression patterns was proposed by D. Hughes and colleagues. They studied interpopulation variability in gene expression in placental tissue from individuals of four populations: Americans of European, South Asian, East Asian, and African ancestry (Hughes et al., 2015). The results indicated that approximately 8 % of gene expression variability was attributable to interpopulation differences, while 58.9 % of transcriptome variability was driven by interindividual differences within a single population. The greatest expression variability was recorded in the African and South Asian populations, where over 140 differentially expressed genes were identified. Genes exhibiting the highest interpopulation variability were predominantly involved in immune response, cellular signaling, and metabolism processes. Despite the strengths of this study, a significant limitation for interpreting the results is the factor of cellular heterogeneity, which cannot be eliminated when using whole tissue. To better understand true variability in gene expression, a study design was proposed in which the transcriptomic landscape was examined in a single cellular subpopulation of decidual cells from individuals of Russian and Buryat populations (Babovskaya et al., 2024). This study was the first to assess intra- and interpopulation variability in genome-wide gene ex- pression at the level of individual placental tissue cells. The findings revealed that interpopulation differences among individuals with physiological pregnancies accounted for 4 %, while interindividual variability contributed 67 % (Nit = 67 %) (Fig. 1). Transcripts involved in regulating apoptotic enzyme activity exhibited the least variability, whereas those participating in renal filtration, blood pressure regulation, TGFβ- mediated processes, and cellular signal transduction showed the greatest variability.

RNA-seq data analysis showed that gene expression variability between populations ranges from 4 to 25 %. However, in studies where total gene expression variability is further divided into components characterizing interindividual and intraindividual variance, this range narrows to 3–8 %. Overall, at these levels, gene expression variability among the studied populations does not, on average, reach the level observed at the genome-wide scale, where the proportion of interpopulation genetic differences in the total genetic diversity of human populations is estimated at 10–15 % (Stepanov, 2016). Nevertheless, the population component significantly influences gene expression variability, underscoring its role in shaping transcriptomic diversity.

**Studies of interpopulation variability in gene expression in disease contexts**

The investigation of polygenic diseases is of significant scientific interest due to their high prevalence across populations. According to epidemiological data, multifactorial diseases (MFDs) account for approximately 90 % of all hereditary pathologies, highlighting their substantial medical and social importance and relevance to modern genetic research (Hoffjan et al., 2016). The risk of MFDs is typically associated with numerous factors, including socioeconomic, demographic, cultural, environmental, and genetic determinants. Our understanding of the genetic determinants of disease risk has expanded considerably with the advent of high-throughput genomic tools, enabling researchers to profile the genome, epigenome, and transcriptome and to broadly analyze the resulting data (Gurdasani et al., 2019; Sirugo et al., 2019). Demographic features, genetic drift, and environmental adaptation over millennia have led to global population differentiation. This genomic diversity opens new opportunities for biomarker discovery, treatment development, and a better understanding of disease risk across populations. Given the accumulated evidence of significant gene expression variability in various pathologies, as well as differences in disease prevalence and clinical manifestations depending on racial background, there is a need to assess the extent to which the population genetic component influences the observed variability.

For example, L. Beretta and coauthors used transcriptomic technologies to study systemic scleroderma in populations of Southern and West-Central Europe, identifying both shared and population-specific pathological pathways in this autoimmune disease (Beretta et al., 2020). The experiment revealed that a key role in the pathogenesis of systemic scleroderma is played by type I interferon activation signaling pathways via TLR receptors, irrespective of population affiliation

The study by Y. Nédélec and colleagues demonstrated population differentiation in infectious diseases. It was found that 9.3 % of genes expressed in macrophages exhibit dif-ferences in regulatory responses to infection linked to population background. Specifically, African ancestry is associated with a stronger inflammatory response and reduced intracellular bacterial growth (Nédélec et al., 2016). However, earlier research indicated that approximately 34 % of genes expressed in macrophages show at least one type of transcriptional divergence related to population affiliation: either differences in gene expression or, less commonly, transcript isoform diversity (Lappalainen et al., 2013). One of the most significant observations by Y. Nédélec and colleagues was the detection of a stronger inflammatory response to infection in individuals of African ancestry. This finding aligns with prior studies showing that Africans more frequently carry alleles associated with heightened proinflammatory responses (Ness et al., 2004) and exhibit elevated levels of circulating C-reactive protein (Kelley-Hedgepeth et al., 2008). Although the precise causal relationship between population affiliation and proinflammatory response remains unestablished, it can be hypothesized that the stronger inflammatory response in individuals of African ancestry explains their enhanced ability to control bacterial growth post-infection (Nédélec et al., 2016).

Cardiovascular diseases have also been a focus of research interest. P. Wei and colleagues examined differential gene expression in endothelial cells between African Americans and Caucasians, exploring the potential contribution of certain genes to observed population differences in the incidence of tumors and cardiovascular diseases. Notably, African Americans (AA) exhibit a 2.4-fold higher incidence and approximately 50 % greater prevalence of hypertension compared to Caucasians (EA). The comparison of AA and EA groups revealed that 31 genes showed differential expression between the two groups at FDR = 0. Four genes exhibited elevated expression in the AA group compared to EA, while 27 genes showed reduced expression in AA (Wei et al., 2011). Among the differentially expressed genes (DEGs), PSPH stood out due to its highest differential expression across the studied groups. Interestingly, a homolog of this gene, PSPHL, was later identified in another study as the most differentially expressed gene between African Americans and Americans of European ancestry. J. Allard and colleagues investigated genes differentially expressed in endometrial cancer between women of Caucasian and African ancestry. It was reported that, in addition to differences in disease incidence, molecular cancer subtypes also vary between carriers from Caucasian and African populations, suggesting the presence of populationspecific features in the expression profiles of tumor tissues. African American women are typically diagnosed with more advanced disease, unfavorable histological types, and higher malignancy grades compared to Caucasians. The study found that gene expression variance between populations accounted for 7.2 % (341 transcripts) at p < 0.005 (Allard et al., 2012). Among these, the phosphoserine phosphatase gene PSPHL was identified as the most hyperexpressed in both tumor and normal endometrial and ovarian tissues of African American women compared to tissues from women of European ancestry. This suggests that PSPHL is not a tumor marker, a conclusion further supported by studies on breast, prostate, and endothelial cells (Wallace et al., 2008; Martin D.N. et al., 2009; Wei et al., 2011).

There is also significant interest in studying populationspecific features of oncological diseases, driven by their substantial contribution to global morbidity and mortality. M. Kinseth and colleagues (2014) established population differentiation in the incidence and progression of prostate cancer. Incidence and mortality rates among African Americans (AA) are 1.5 and 2.3 times higher, respectively, than among individuals of Caucasian ancestry. AA also tend to experience more aggressive disease progression and earlier onset. Notably, this study again demonstrated differential expression of the phosphoserine phosphatase genes (PSPH) and CRYBB2 between individuals of European and African ancestry (Wallace et al., 2008). Consistent with prior research, the authors concluded that these genes are not associated with tumor tissue but may serve as markers of racial background (Kinseth et al., 2014).

Another study on population differentiation in gene expression in oncology, conducted by K.A. Mitchell and colleagues, aimed to determine whether racial differences in gene and microRNA expression influence clinically significant differences in lung tumor biology between African Americans (AA) and Americans of European ancestry (EA). They showed that while there are similarities in expression profiles between the populations, differences exist in both protein-coding transcripts and the non-coding genome. The researchers found that the transcriptome of AA tumor cells was enriched in stem cell and invasion pathways, whereas the transcriptomic profile of EA tumor cells showed enrichment in categories related to cell cycle, mitosis, and proliferation processes. The authors noted hypoexpression of the genes ARL17A, LRCC37A3, and KANSL1 in lung tissues of AA compared to EA (Mitchell et al., 2017). These genes are located in the 17q21 region, where structural diversity – an inversion polymorphism with duplication – has been previously identified (Steinberg et al., 2012). The presence of a direct (H1) or inverted (H2) haplotype influences differential susceptibility to non-allelic homologous recombination and diseases, including cancer. European populations exhibit a high frequency of duplication events, whereas most West African populations lack those (Steinberg et al., 2012). Other studies on breast, colon, prostate, and endometrial tissues have also identified genes with expression varying by population affiliation, such as PSPHL, CRYBB2, AMFR, and SOS1 (Martin D.N. et al., 2009; Allard et al., 2012; Field et al., 2012; Mitchell et al., 2017).

Alongside oncological diseases, reproductive health holds high social significance. However, studies of the transcriptome in reproductive system disorders using a population-based approach remain rare. This is due to both limited availability of biological samples and methodological challenges, such as the need to account for hormonal fluctuations and other factors affecting gene expression. Nevertheless, such studies are critical for understanding the etiology of reproductive disorders and developing personalized treatment approaches. Previous research has demonstrated racial and ethnic differences in the incidence of preeclampsia (PE), a severe pregnancy complication associated with high maternal and infant mortality. For instance, studies by Torchin, Ghosh, and Wolf indicate that African American women have a higher risk of developing PE compared to women of European ancestry (Wolf et al., 2004; Ghosh et al., 2014; Torchin et al., 2015). Interpopulation variability in the expression levels of genes and proteins playing key roles in PE pathogenesis may contribute to these differences. For example, A. Whitney and colleagues showed that average levels of prognostic angiogenic (PlGF) and antiangiogenic factors (sEng, sVEGFR1) differ significantly between African Americans and Caucasians living in the United States (Whitney et al., 2003). Notably, both domestic and international studies have reported associations between polymorphic markers of genes encoding angiogenic and antiangiogenic factors and the risk of preeclampsia (Akulenko et al., 2020; Rana et al., 2022). These findings highlight the importance of studying the genetic aspects of preeclampsia while considering population-specific characteristics.

**Fig. 2.**
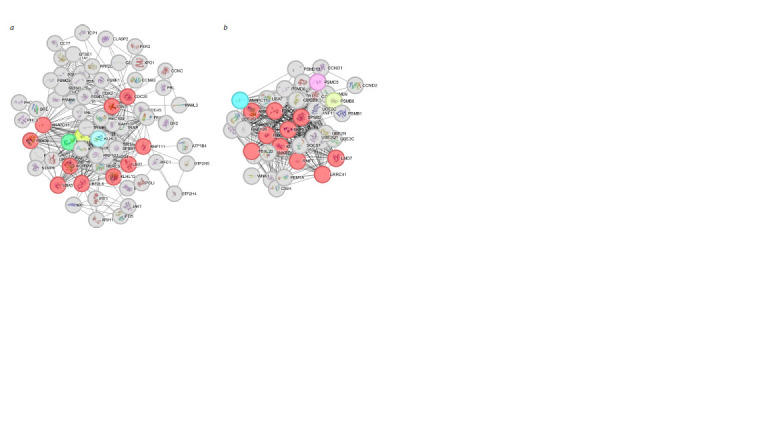
Top 10 hub genes (red) with their nearest neighboring nodes (gray) in the protein-protein interaction network: a – Russians, b – Yakuts.

In Russia, one of the first studies aimed at identifying molecular mechanisms underlying severe pregnancy complications, such as preeclampsia, while accounting for the genetic features of Siberian populations, was conducted using highdensity microarrays. This study included genome-wide gene expression profiles from 21 women of Russian (European) and 23 women of Yakut (East Asian) ancestry (Trifonova et al., 2022). The study demonstrated a bioinformatics approach to data analysis that not only identified differentially expressed genes between populations but also analyzed clusters of coexpressed genes within protein-protein interaction network models and highlighted functionally active hubs (hub genes) in these networks. Network analysis of co-expression gene blocks yielded 10 clusters containing 7,968 genes associated with PE in Russians and 9 clusters containing 7,966 genes in Yakuts. Construction of protein-protein interaction networks identified the most functionally active genes: CUL1, ANAPC11, LNX1, CDC20, UBE2L6, FBXO9, KLHL13, UBA3, KCTD7, RNF111 in Russians (Fig. 2a), and KLHL3, FB11PSXL, ASB2, LRRC41, LMO7, RNF7, SKP2, FBXO2 in Yakuts (Fig. 2b). These genes are predominantly involved in the assembly and regulation of the ubiquitin-ligase complex. In total, 1,701 genes associated with PE were common to both populations. Functional annotation of these genes revealed their involvement in interferon activity and ubiquitin-ligase complex function (Trifonova et al., 2022).

## Prospects for applying transcriptome research in forensics

The evidence presented above regarding the existence of interpopulation variability in gene expression across different human populations, along with the identification of specific markers characteristic of certain population groups, suggests that this knowledge could complement standard protocols used in forensic science (Daca-Roszak, Zietkiewicz, 2019).

Transcriptomic data could serve as an additional tool for individual identification, particularly in cases where traditional methods, such as short tandem repeat (STR) analysis, are insufficiently informative due to DNA degradation. Studies demonstrate that gene expression exhibits high individual specificity, driven by both genetic variations and epigenetic modifications (Spielman et al., 2007; Storey et al., 2007; Stranger et al., 2007; Price et al., 2008; Zhang et al., 2008; Fan et al., 2009; Lappalainen et al., 2013). These findings indicate that carefully selected population-specific transcriptomic markers could be utilized in forensics similarly to DNA-based marker methods to infer the population origin of forensic samples, especially when standard protocols are hindered by sample heterogeneity (e. g., mixed samples).

In practice, identifying multiple contributors through DNA marker genotyping in forensic samples is challenging or infeasible when reference DNA profiles are unavailable (Westen et al., 2009). Furthermore, in conjunction with identity determination, population-specific markers could be used to accurately estimate the time of death. Results from using RNA analysis as a complement to the forensic toolkit show that gene expression patterns change post-mortem in a tissue- and individual-specific manner, potentially allowing for the estimation of the time of death (González-Herrera et al., 2013; Ferreira et al., 2018). However, successfully integrating transcriptomic markers into forensic investigations requires further research, standardization of protocols, and the development of specialized databases

## Conclusion

Population transcriptomics studies represent a powerful tool for analyzing the genetic and molecular mechanisms underlying complex phenotypic traits. These studies enable the identification of population-specific features in the transcriptomic profiles of cells and tissues, which can be applied both for fundamental scientific purposes and in practical contexts such as forensic expertise, including biomarker analysis and genetic sample identification. Moreover, population transcriptomics opens new avenues for understanding the molecular mechanisms of diseases across diverse human populations. Recent research has demonstrated that genetic and transcriptional differences between populations may play a key role in the pathogenesis of autoimmune, infectious, oncological, and reproductive system diseases. Further advancements in population transcriptomics could lead to the development of novel approaches for the diagnosis, prevention, and treatment of diseases, tailored to the population-specific characteristics of individuals. Thus, population transcriptomics emerges as a promising field in molecular biology, capable not only of deepening our understanding of the genetic and epigenetic mechanisms underlying phenotypic differences between populations but also of providing a scientific foundation for implementing innovative strategies in medical practice.

## Conflict of interest

The authors declare no conflict of interest.
